# Albert Kligman, also a Hair Man

**DOI:** 10.4103/0974-7753.66923

**Published:** 2010

**Authors:** Shyam B Verma

**Affiliations:** Consultant Dermatologist Gujarat, Hon. Dermatologist to H.E. The Governor of Gujarat State, India, Clinical Associate Professor, University of Virginia, Charlottesville, Clinical Assistant Professor, SUNY at Stony Brook, USA

**Figure 1:**
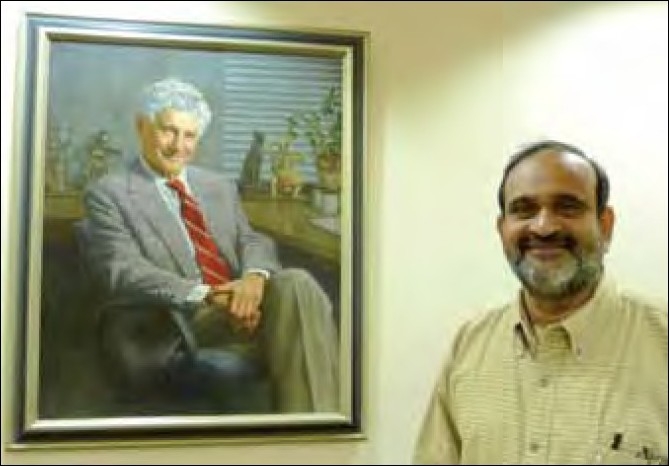
Portrait of Prof. Albert Kligman in the Department of Dermatology, University of Pennsylvania Hospital, Philadelphia, USA

The world lost a legend in dermatology on 9^th^ February 2010. Albert Kligman [Figure 1], the iconic professor of dermatology, left us at the age of 93 years (1916–2010). Albert Kligman does not need much of an introduction. Our community knows of him by his path-breaking research and invention of the molecule “tretinoin,” which he discovered and patented first for the treatment of acne and later for photoaging. Prof. Kligman, in his larger-than-life career spanning over 60 years, is known for his research and expertise in acne, rosacea, contact dermatitis and cutaneous toxicology amongst others. Because this obituary appears in the International Journal of Trichology, it would not be complete without mentioning his keen interest in hair! Many will be surprised to know that common phenomena and terms like the “human hair cycle,” “telogen effluvium” and “hot comb alopecia” were introduced by him in 1959, 1961 and 1968, respectively. He was the author of dozens of books, hundreds of scientific papers and a brilliant, witty and erudite speaker. According to many of his students, who are now dermatologic icons in their own right, he was a great teacher and loved to stimulate discussion on all matters dermatologic. Like many confident and brilliant people, he was at times perceived to be arrogant with a penchant for making critical remarks with candor, a trait that understandably put him a notch lower than many in popularity, but he spoke his mind nevertheless. Also, like many famous people, he was hounded for the smallest crack that could be seen in his dermatologic career, which many envied. He got bad press for the “infamous” experiments that he conducted in an American prison on inmates and the press hounded him probably much more than what was required. That was the price he paid for being famous.

I cannot claim to be his friend, but I had interacted with him on a few occasions. He was fascinated by India. We were lucky to have him and his wife in Mumbai during one of the earlier CSI conferences. Then, I met him a couple of times in the University of Pennsylvania during one of my lectures, where I now serve as a Clinical Associate Professsor. He was attending the grand rounds, looking old and fragile, but his eyes reflected his enthusiasm and alertness. He knew some of the old icons of Indian dermatology, like Dr. Sharat C. Desai. He had encouraged people like Prof. B. S. Verma, another old timer who met him in St. John’s Institute of Dermatology in London at a mycology laboratory while doing his doctorate in, the early 60s. Dr. Verma reminisced that he was exceptionally brilliant in understanding what was shared with him in a matter of minutes. He actively encouraged the younger generation, a forward-looking approach and a sign of maturity. His students vouch for his sense of humor, wit and generosity. All this made him one of the brightest shining stars in the dermatologic firmament. He will continue to shine, if not in his presence, in his work, which will not be forgotten for centuries. Dr. Guy Webster at Jefferson University in Philadelphia rightly called him “Father of Modern Dermatology.” May God bless his soul. And, may we always emulate his enthusiasm for research and dedication to dermatology.

